# Kinematics of anterior cruciate ligament-deficient knees in a Chinese population during stair ascent

**DOI:** 10.1186/s13018-016-0423-9

**Published:** 2016-08-08

**Authors:** Chang Zhao, Chuangxin Lin, Wenhao Wang, Chun Zeng, Hang Fang, Jianying Pan, Daozhang Cai

**Affiliations:** Department of Orthopedics, Academy of Orthopedics, Guangdong Province, The Third Affiliated Hospital of Southern Medical University, 183 Zhongshan Avenue West, Guangzhou, 510665 China

**Keywords:** Anterior cruciate ligament, Knee, Gait, Kinematics, Chinese

## Abstract

**Background:**

The purpose of this study was to measure the tibiofemoral kinematics of anterior cruciate ligament (ACL) deficiency in a Chinese population and compare the kinematics with published data about a Caucasian population.

**Methods:**

Unilateral knees of 18 Chinese ACL-deficient (ACL-D) subjects were studied while subjects ascended stairs. Kinematic alteration was compared between ACL-D knees and contralateral ACL-intact (ACL-I) knees. The kinematic alteration of ACL deficiency was also compared between the Chinese population and published data about a Caucasian population.

**Results:**

A statistical difference was found in the three-dimensional rotations between ACL-D and ACL-I knees. In the sagittal plane, ACL-I knees had a larger flexion angle than ACL-D knees during 40 to 50 % of the activity during stair ascent (*P* < 0.027) and throughout the gait cycle. A significant difference in rotational motion between ACL-D and ACL-I knees was also observed in the frontal plane during 40 to 60 % (*P* < 0.017) of the activity and in the transverse plane during 70 to 80 % (*P* < 0.028) of the activity. A greater tibial varus was demonstrated in the Chinese population while the published data revealed external tibial rotation and a statistical difference in translation in the Caucasian population.

**Conclusions:**

ACL-D knees show different kinematics than ACL-I knees in the Chinese population. ACL-I knees had a larger flexion angle than ACL-D knees in the middle stage of the activity during stair ascent. A significant difference in rotational motion between ACL-D and ACL-I knees was also observed in the frontal plane during the middle phase and in the transverse plane during the terminal phase of the activity. A greater tibial varus was demonstrated in the Caucasian population while the published data revealed external tibial rotation and a statistical difference in translation in the Caucasian population.

## Background

Ascending stairs is a common activity in daily life and has been adopted as a closed-kinetic chain exercise in various lower extremity rehabilitation protocols [24]. When attempting to stabilize their knees while stepping up, patients with anterior cruciate ligament (ACL) injury exhibit altered tibiofemoral kinematics, knee joint moment, muscle co-activation, shear forces, and ACL strain [13]. Therefore, understanding the adaptations that patients with ACL deficiency employ during stair climbing is useful for not only assessing the patients’ ability to manage the injury with respect to potential for joint complications but also optimizing the rehabilitation protocol in order to enhance its efficacy in ACL reconstruction and total knee arthroplasty, and for treating different pathologies of the knee, such as osteoarthritis (OA) [32].

Some research studies pertained to activities such as ascending stairs. For instance, in a recent study, ACL-D knees demonstrated significantly increased anterior tibial translation, medial tibial translation, and external tibial rotation [17]. Gao et al. investigated the three-dimensional (3D) joint kinematics of ACL-D and ACL-reconstructed knees during stair ascent and descent and found that the ACL-D knees exhibited significant extension [11]. However, they did not investigate ACL-D knees in subjects with concomitant injuries, such as meniscus injuries, collateral ligament injuries, and cartilage degeneration. Although these studies have greatly improved our knowledge of knee kinematics during step-up activities, the different experimental designs and coordinate system selections make it difficult to obtain a systematic understanding of the knee joint kinematics during step-up activities.

Therefore, the purpose of the present study was to elucidate the gait of patients with ACL deficiency with or without combined medial or lateral meniscus tear during stair ascent in order to determine the effects of ACL deficiency on knee joint motion during step-up activities, including the six degrees of freedom (6DOF) at the knee. Specifically, all subjects included were of Han nationality, which is the largest ethnic group in the Chinese population. We employed an established and validated technique utilizing single-plane magnetic resonance imaging, single-plane fluoroscopic imaging, and a computer model that can measure knee kinematics during unrestricted dynamic motion with high accuracy [9]. We hypothesized that during the single-leg step-up activity, the ACL-D knee would show significantly different kinematics than those of uninjured contralateral knees.

## Methods

### Subject recruitment

Eighteen Chinese subjects with unilateral ACL-D knees, ranging in age from 19 to 43 years (12 men and 6 women, average body mass index, 23.9 ± 2.2 kg/m^2^), were recruited for this study. ACL injury was documented via MRI and clinical examination (e.g., anterior drawer test, Lachman test, pivot shift test, medial/lateral stress test, McMurray test). The inclusion criteria included patients with confirmed unilateral ACL-D knees based on intra-operative findings. The exclusion criteria included ACL-D subjects with any knee disorders, symptoms, or anatomical abnormalities. The exclusion criteria also included ACL-D subjects who had a history or evidence of injury, surgery, or disease in their contralateral knees. Subjects were also evaluated for the absence of abnormal motions of the hip and ankle joints when ascending stairs. Approval of the experimental design by the authors’ institutional review board was obtained prior to the initiation of the study. A signed consent form was obtained from each subject before any testing was performed.

### Creation of 3D knee model

The knee joint segments of each subject were scanned using computed tomography (CT, SOMATOM Definition; Siemens, Munich, Germany). Parallel digital images with a thickness of 1 mm without a gap and with a resolution of 512 × 512 pixels were obtained. The images were then imported into solid modeling software Mimics 17.0 (Materialise, Leuven, Belgium) and manually digitized in order to outline the contours of the femur and tibia. These outlines were used to construct 3D geometric models of the knees.

### Measurement of in vivo knee kinematics

**Fig. 1:**
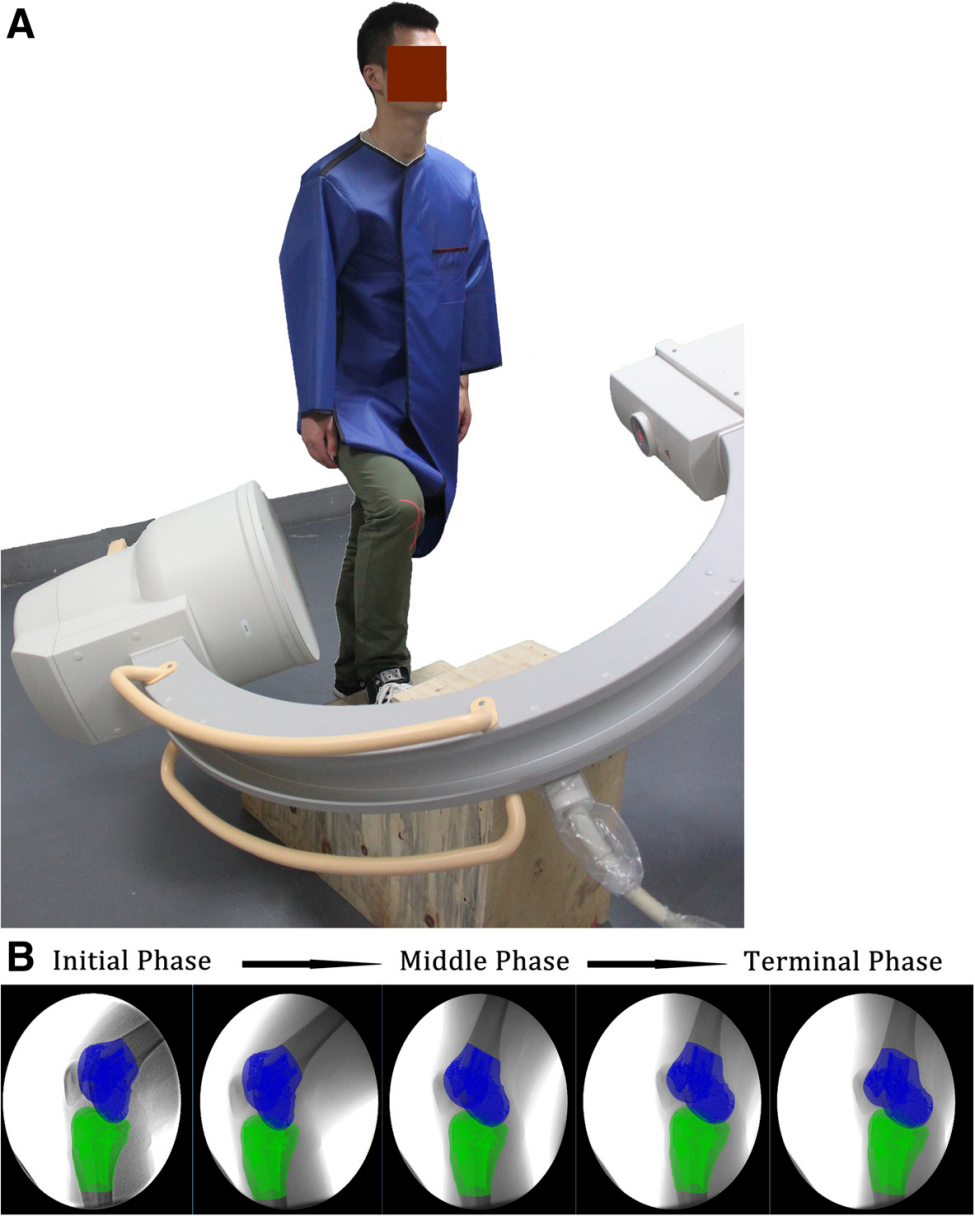
**a** Measurement of in vivo knee kinematics during ascending stairs by single fluoroscopic imaging system. **b** Virtual reproduction of tibiofemoral kinematics during ascending stairs

A single-plane fluoroscopic imaging system that was previously validated for treadmill gait analysis was used to determine the 6DOF kinematics of both the injured knees and intact knees during stair ascent [34]. Laser-positioning devices that were attached to fluoroscopes helped to align the target knee within the field of view of the fluoroscopes while subjects ascended the stairs (Fig. 1a). Each subject was asked to walk up a custom set of stairs. Each step was 18 mm high, 20 mm deep, and 40 mm wide. The dimensions of the stairs were designed to be similar to those found in most buildings in Singapore and were within published ergonomic recommendations [16]. The postures of subjects were carefully examined under the direction of an orthopedic surgeon in order to reduce variation. No constraint was applied to the knees of the subjects while they performed active motions. Subjects were allowed to ascend the stairs at a self-selected pace, and a rhythmic alarm was used to help the patients ascend the stairs at a fixed pace. The entire experiment took approximately 10 min to complete, and images were processed in the Digital Imaging and Communications in Medicine format.

Fluoroscopic images of the knee were captured at a specific posture, and comma-separated value files were imported into the registration software, Virtual_knee1.0 (Medmotion, Guangzhou, China). The actual positions of the image intensifiers of the fluoroscopes were then reproduced (Fig. 1b). A virtual camera was created inside the virtual space in order to reproduce the positions of the X-ray sources with respect to the image intensifiers. Therefore, the geometry of the single-plane fluoroscopic system was recreated in the solid modeling program. The CT image-based 3D knee models were introduced into the virtual fluoroscopic system and viewed from the perspective views of the single-plane fluoroscopic camera. These models could be independently translated and rotated in 6DOF until their outlines matched the osseous outlines captured on the single-plane fluoroscopic images. This process was executed using an established protocol [34]. The software (Virtual_knee1.0) allowed the models to be manually translated and rotated in increments of 0.2 mm for in-plane translation and 3.25 mm for out-of-plane translation, with an accuracy of 1.57° for rotation in a knee. Manual matching was first performed. This was followed by an automated matching process. As a part of this technique, the knee positions during in vivo weight-bearing activities were reproduced, representing the 6DOF kinematics of the knee for each in vivo posture.

**Fig. 2:**
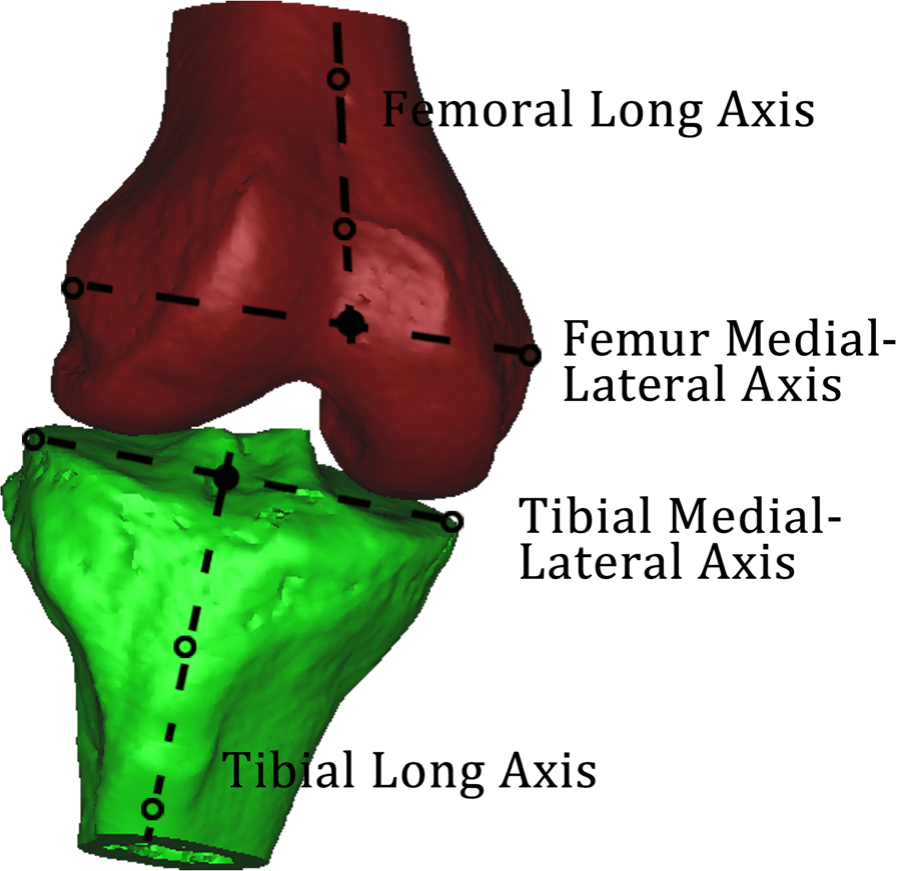
Definition of local femur and tibia coordinate systems. In the femur, the first two points were the prominent points of the medial and lateral femoral epicondyles. The other two points were located paralleling to the wall of the femur shaft. The transepicondylar line was obtained by linking the most pivot points on the medial and lateral condyles. The femoral origin was located at the midpoint of the transepicondylar axis. The line that is parallel to the shaft of the femur was defined as the long axis of the femur. In the tibia, the first two points were the most pivot points on the medial and lateral tibia plateau. The other two points were located paralleling to the wall of the tibia shaft. The *line* connecting to the most pivot points on the medial and lateral tibia plateau was defined as the medial-lateral axis, and the *midpoint* of this line was defined as the origin of the tibial coordinate system. The *line* that is parallel to the shaft of the femur was defined as the long axis of the femur. Tibiofemoral rotation and translation was defined as the motion of the femoral center move with respect to the origin in the tibial coordinate system

A consistent coordinate system was used in order to estimate the kinematics of both knees of each subject based on the series of matched bone models (Fig. 2). Because the same coordinate system was used for both the ACL-I and ACL-D knees, we were able to reduce the variability of our measurements caused by differences in coordinate systems. Specifically, we imported the 3D model binary stereolithography file from Mimics 17.0 into Geomagic studios 2014 reverse modeling software (Geomagic, Morrisville, North Carolina, USA) and obtained four points. We employed this “four-point” method to build coordinate systems in the femur and tibia. In the femur, the first two points were the prominent points of the medial and lateral femoral epicondyles. The other two points were located parallel to the wall of the femur shaft. In the tibia, the first two points were the most external points on the sides of the medial and lateral tibia plateau. The other two points were also located parallel to the wall of the tibia shaft. This is a convenient method for investigators to build custom coordinate systems. We repeated the process for each 10 % of the activity from the beginning until the end of weight-bearing.

### Statistical analysis

A two-way repeated measure analysis of variance was used to compare the tibiofemoral kinematics of the ACL-I and ACL-D knees. The two within-subject factors were knee status (ACL-D vs. contralateral knees) and time point (every 10 % of the ascending phase). The level of statistical significance was set as *P* < 0.05. When a statistically significant difference was detected, a post hoc pairwise comparison was performed, and the level of statistical significance for this was also set as *P* < 0.05. The statistical analysis was performed using commercially available software (SPSS for Windows 13.0, Chicago, IL, USA).

## Results

**Fig. 3:**
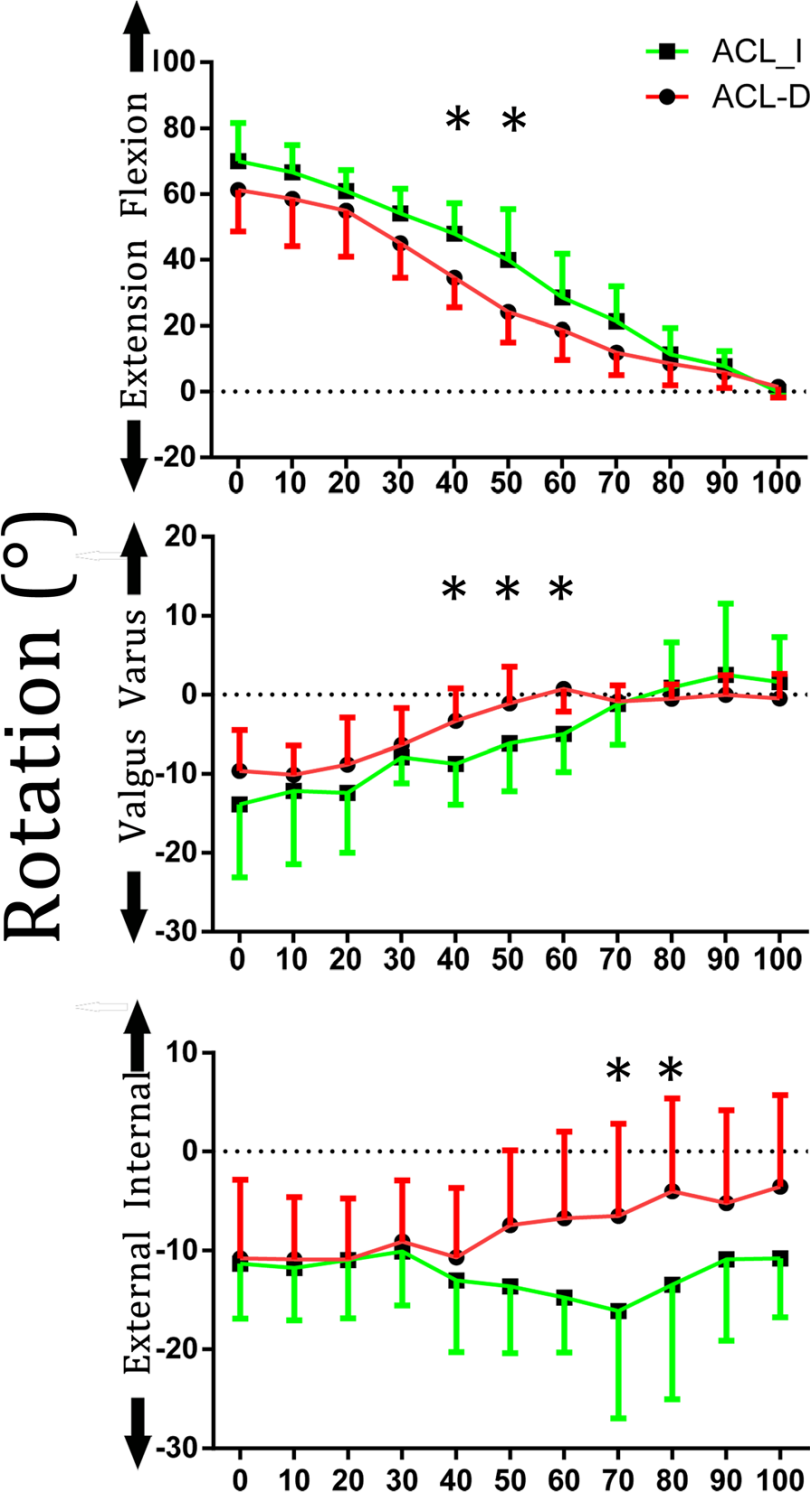
Tibiofemoral kinematics (rotations) of healthy and ACL-deficient knees during ascending stairs. The values represent the motion of the femur with respect to the tibia. *Asterisk* denotes statistically significant difference at *P* < 0.05

Primary rotation averaging 70° occurred in the sagittal plane (flexion-extension) (Fig. 3). The secondary rotations in other rotational planes had much smaller amplitudes (averaging 4° to 10°). From the beginning to the end of stair ascent, the flexion angle consistently decreased from an average of 70° at 0 % to an average of 1° of hyperextension at 100 % of activity progress. ACL-I knees had larger flexion angles than the ACL-D knees during the time from 40 to 50 % of the activity during stair ascent (47.9 ± 9.2° vs. 34.5 ± 9.0°; 39.9 ± 15.5° vs. 24.1 ± 9.2°, *P <* 0.027) and throughout the ascent. A significant difference in rotational motion between the two knee conditions was observed in the frontal plane at 40 to 60 % (−6.3 ± 4.6° vs. −7.9 ± 3.2°, −3.3 ± 4.1° vs. −8.7 ± 5.1°, −1.0 ± 4.6° vs. −6.1 ± 6.1°, *P* < 0.017) and in the transverse plane at 70 to 80 % (−6.4 ± 9.3° vs. −16.1 ± 10.8°, −3.9 ± 9.3° vs. −13.4 ± 11.5°, *P* < 0.028) of the activity. From the initiation of weight-bearing until 60 % of the stair ascent was completed, ACL-D knees displayed an average of 3° of extra varus tibial rotation. Substantial differences were also found in the transverse plane. ACL-D knees exhibited 3° to 5° more internal rotation than ACL-I knees during the final 60 % of the activity.

**Fig. 4:**
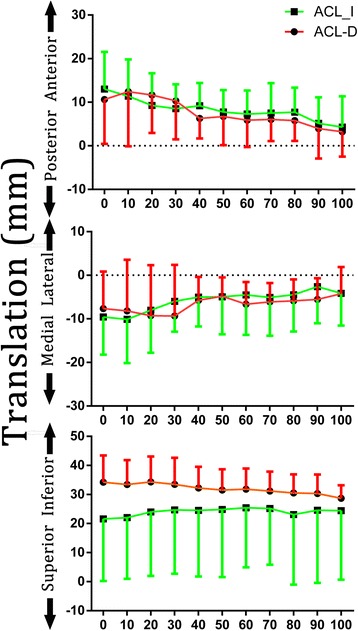
Tibiofemoral kinematics (translations) of healthy and ACL-deficient knees during ascending stairs. The values represent the motion of the femur with respect to the tibia. *Asterisk* denotes statistically significant difference at *P* < 0.05

With respect to translational motion, a statistically significant difference was not found in translation (Fig. 4). During the first 10 to 30 % of the activity, the ACL-D knees had larger anterior tibial translation than ACL-I knees (12.4 ± 12.5 vs. 11.3 ± 8.4 mm; 11.6 ± 8.6 vs. 9.2 ± 7.4 mm; 10.3 ± 8.85 vs. 8.53 ± 5.5 mm). No statistical difference was observed between the two conditions. Although ACL-D knees exhibited 3–13 mm more inferior translation, no significant difference was found during the activity.

## Discussion

Tibiofemoral kinematics during stair ascent is investigated in patients with ACL-D knees and uninjured contralateral knees using CT, dynamic single-plane fluoroscopy, and a semi-automated matching technique. The initial hypothesis is confirmed because ACL-D knees show different kinematics than ACL-I knees among the Chinese population. In particular, ACL-I knees have larger flexion angles than ACL-D knees at the middle stage of the activity of stair ascent. Reduced valgus is also observed in ACL-D knees during the middle phase and in the transverse plane during the terminal phase of the activity. Greater tibial varus is demonstrated in the Caucasian population while published data reveal external tibial rotation and a statistical difference in translation in the Caucasian population.

ACL deficiency has been shown to disturb the flexion-extension motion during stair climbing [11]. Previous research studies found smaller flexion angles and moments for ACL-D knees than in the current studies [31]. Some research studies reported that the peak flexion moment of the involved limbs of patients was significantly smaller than those of the uninvolved limbs and control limbs by up to 50 % [29]. Additionally, another study found that reduced knee extension moments, which resist flexion moments, are exhibited in patients with reconstructed ACLs [14]. Knee extension moments are indicative of the neuromuscular function of the quadriceps and hamstrings [15]. In our study, the ACL-I knees exhibit larger flexion angles than ACL-D knees in the middle phase of the activity during stair ascent and throughout the gait cycle. Because the dominant effect of the ACL is to restrain anterior tibial translation, ACL-D subjects appear to use compensation strategies, whereby the quadriceps reduces flexion angles during a functional movement in order to reduce the challenge of the motor task [29]. It is widely believed that a spatial shift in the location of load contact will lead to the degeneration of the articular cartilage, but the clinical relevance of these small alterations in knee flexion angles remains unclear [19]. Moreover, results from other studies are inconsistent. Previous studies claimed that statistical differences in flexion-extension are found in the terminal stage during stair ascent [11]. Some studies found no statistical difference in flexion-extension [17]. These discrepancies between results may have resulted from insufficient statistical power, differences in the study subjects or testing protocol, and different coordinate systems used in the analyses. These differences in subject background and design likely affect the knee kinematics in the axial plane.

In addition to flexion-extension, altered kinematics is also found in the frontal and transverse planes. ACL-D knees exhibit offsets in varus and internal tibial rotation. These results are concordant with kinematic abnormalities found in some experiments [11, 28]. A similar trend in the internal tibial rotation has been reported by some researchers for ACL-D and ACL-reconstructed knees during level walking [1]. The magnitudes of the offset in the frontal rotation are larger than those in axial rotation, and they are consistent throughout most of the activity. It is proposed that altered muscle coordination is probably essential in ACL-D patients in order to secure knee stability. Bulgheroni et al. [7] reported a reduction in quadriceps activity while Beard et al. [4] found increased hamstring activity in ACL-D patients. Whether it is increased hamstring or reduced quadriceps activity, the net result of both conditions is reduced flexion moment, suggesting an inhibition of quadriceps function. As a result, there is greater tibial internal rotation. Similar results were found by some research studies [5]. Namely, individuals with ACL deficiency exhibit greater knee internal rotation during higher demand activities, such as ascending and descending steps or jogging. With a more varus position, the medial compartment of the knee joint tends to be more compressed. With greater tibial internal rotation, the contact location on the medial compartment of the tibia plateau could shift anteriorly while the contact on the lateral compartment could shift posteriorly. Such abnormal kinematics is likely responsible for the degeneration of articular cartilage in the knee joint, especially within the medial compartment [2]. Clinical studies have shown that ACL-D patients are more vulnerable to the development of osteoarthritis in the medial compartment of the knee [26]. Moreover, a greater internal rotation moment is found in knees with moderate OA compared to asymptomatic knees during gait or other activities [3].

A statistically significant difference is not found in translation. Some researchers found that translation during step ascent and descent does not differ between injured and control knees, which is similar to our findings [31]. The authors explained that a compensatory mechanism through the action of muscular co-contraction substituted for the ACL deficiency. However, one study found a 2.5-mm difference, on average, in anteroposterior translation between the conditions [17] and suspected that ascending stairs or stepping up would likely introduce microtrauma to the cartilage with potentially deleterious consequence by altering the contact stress distribution [30]. The discrepancies between these studies can be attributed to differences in coordinate systems, testing protocol, and the method used to determine knee kinematics. For example, some studies used the geometric central axis and transepicondylar axis coordinate system [18], and a four-point system is used in the present study. This difference may have affected the translation kinematics.

Compared to other studies in which subjects of different populations performed stair ascension, our study demonstrates a different pattern of kinematics (Table 1). However, some small differences still exist among different studies due to protocols such as the designs of stairs, the variety of coordinate systems, and so forth. Greater tibial varus is observed in the Asian population while external tibial rotation and a statistical difference in translation are found in the Caucasian population. Except for different protocols, these differences are attributed to the differences in the anatomy of the intercondylar notch, mechanical axis, and tibiofemoral alignment [33]. A higher quadriceps angle (Q-angle), varus alignment, and abnormal lower limb mechanical axis, including knee recurvatum, excessive navicular drop, and excessive subtalar pronation, are anatomic malalignments related to increased risk of ACL injury [21]. A higher Q-angle places the knee at risk of static and dynamic valgus stress [23]. The lower limb alignment is more varus, and the knee is medially inclined in the Chinese population when compared to the Caucasian population [33]. Our observations of greater tibial varus are compatible with these findings.

**Table 1:** Kinematic alteration of ACL-D patients by race

Study	Race	Main kinematic alteration
Kozánek et al. [17]	Caucasian	Greater anterior/medial tibial shift; greater external tibial rotation
Gao et al. [11]	Caucasian	Greater varus and internal tibial rotation
Vergis et al. [31]	Caucasian	Greater anterior tibial shift; no significant rotation
Takeda et al. [28]	Asian	Greater tibial varus/external rotation
This study	Chinese	Greater tibial flexion/varus/internal rotation

In addition to an abnormal lower limb mechanical axis, tibiofemoral alignment, and the Q-angle, the intercondylar notch width may also contribute to different risks of ACL injuries and patterns of kinematics among people of different races [6]. Previous studies [10] have shown that patients with small intercondylar notches have smaller ACLs and are more susceptible to ACL injury. Another study reported that the notch width in the Chinese population is larger than that in Western populations [8]. This may be due to differences in body size and height and the methods used to obtain tunnel radiographs. Some available evidence concludes that African Americans have significantly statistically wider intercondylar notch widths on 45° flexed weight-bearing posteroanterior radiographs than Caucasians of the same gender [27]. We can speculate from these studies that the anatomy of the intercondylar notch may be significantly different among people of races (Table 2). As a result, these morphologic differences lead to different kinematics between Chinese and Caucasian populations.

**Table 2:** Intercondylar notch width by race

Study	Race	Intercondylar notch width (mm)
Chuang et al. [8]	Chinese	21.23 ± 2.81
Shelbourne et al. [27]	Caucasian	16.9 ± 3.1 (9–27)
Shelbourne et al. [27]	African American	18.0 ± 3.6 (10–27)

Nonetheless, others found that these factors are not predictive of ACL injury risk [23]. The specific role of these factors warrants further research.

The motion analysis method used in this study is single-plane fluoroscopy. It is found to be more accurate than the optical marker-based motion system [12]. Single-plane fluoroscopy provides a less restricted field of view than dual-plane fluoroscopy, and it allows patients to perform dynamic activities more naturally. An optimization algorithm is introduced in order to analyze data and for semi-automated 2D–3D registration, which makes registration efficient.

This study has a number of limitations. First, the number of samples (18 subjects) is relatively small. Moreover, only the Han race is included, so the results cannot be generalized to all races in China. Second, instead of asymptomatic knees from healthy subjects, we use the uninjured contralateral knees as the control group, which may not represent normal function [25]. Third, the accuracy of 2D–3D registration methods using single-plane fluoroscopy is poor for out-of-plane (i.e., mediolateral) translations [22]. ACL deficiency has been shown to affect tibial mediolateral translation in studies utilizing bi-plane imaging techniques. Although bi-plane techniques provide smaller measurement errors, as previously mentioned, single-plane methods provide a less restricted field of view and allow patients to perform dynamic activities more naturally. Fourth, despite the use of a coordinate system that would be convenient for clinicians to use, results may be incomparable to those of other studies because the selection of different knee coordinate systems results in different descriptions of the knee kinematics [20]. Our data indicate that the condylar motion might be different if a different flexion axis is selected. Fifth, we did not measure body mass index (BMI) or muscle strengths, which are likely to have impacts on results.

## Conclusions

In the Chinese population, ACL-I knees had a larger flexion angle than ACL-D knees in the middle stage of the activity during stair ascent. Greater tibial varus was demonstrated in the Caucasian population while the published data revealed external tibial rotation and a statistical difference in translation in the Caucasian population. The differences in kinematics between different populations may provide insight into the enhancement of race-based surgical approaches in order to adjust racial variety.

## Abbreviations

ACL, anterior cruciate ligament; ACL-D, ACL-deficient; ACL-I, ACL-intact; OA, osteoarthritis; 6DOF, six degrees of freedom
